# Experiences of peer support workers supporting individuals with substance use disorders in Egypt: phenomenological analysis

**DOI:** 10.1186/s12913-022-08393-5

**Published:** 2022-08-08

**Authors:** Nashwa Ibrahim, Abeer Selim, Fiona Ng, Muhamed Kasaby, Amira Mohammed Ali, Rasha Eweida, Doha Almakki, Amna Elaagib, Mike Slade

**Affiliations:** 1 Psychiatric and Mental Health Nursing Department, Faculty of Nursing, Mansoura University, Mansoura, Egypt; 2 College of Nursing, King Saud Bin Abdulaziz University for Health Sciences, Riyadh, Saudi Arabia; 3 King Abdullah International Medical Research Center, Riyadh, Saudi Arabia; 4 School of Health Sciences, Institute of Mental Health, University of Nottingham, Nottingham, UK; 5 Center for Global Mental Health, London School of Hygiene and Tropical Medicine, London, UK; 6 Department of Psychiatric Nursing and Mental Health, Faculty of Nursing, Alexandria University, Alexandria, 21527 Egypt; 7 MBBS AlMughtaribeen University, Khartoum, Sudan; 8 Nord University, Postboks 474, 7801 Namsos, Norway

**Keywords:** Peer support workers, Substance use disorders, Low-Middle Income Countries, Qualitative Research, Recovery

## Abstract

**Background:**

Peer support work for substance use disorders is widely implemented in high-income countries. More research is still needed to understand its applicability in settings which have proportionately low budgets allocated to mental health. Peer Support Workers are individuals who managed to achieve recovery from substance use disorders and help people remain engaged in their recovery and prevent relapse through shared understanding.

**Aim:**

To investigate the experience of peer support workers providing recovery support to people with substance use disorders in Egypt.

**Methods:**

A qualitative phenomenological design was used in which 17 adults working as peer support workers for substance use disorders were recruited by means of purposive and snowball sampling. A semi-structured interview with participants was conducted by phone or video-call. Interviews were transcribed and thematically analysed based on descriptive phenomenology.

**Results:**

Three superordinate themes were identified: role responsibility, Peer Support Workers’ need for organizational and stakeholders’ support, and challenges to the role integrity.

**Conclusion and recommendations:**

The findings indicate the need for national and governmental support to peer support workers engaged with people with substance use disorders in Egypt and educating families and the public about the role of peer support workers in substance use disorders.

## Background

According to the latest statistics about regular drug use in Egypt, prevalence rates in Cairo are 33%, 9.6% in Delta region, and 22.4% in Upper Egypt [1]. The situational analysis and response of drug use and its harms in the Middle East and North Africa revealed growing trends relating to Human Immunodeficiency Virus infection (HIV) in Egypt (42%), and Egypt reported the highest prevalence rate of Hepatitis C Virus (HCV) in the world (15–20%) [2].

The health care system in Egypt is diverse in nature and is predominantly financed by out-of-pocket payments [3]. In public mental health settings, addiction treatment units exist within psychiatric hospitals or as a stand-alone specialized addiction treatment hospital e.g. Al-Matar Public Addiction Hospital. Non-profit initiatives exist in Egypt for treatment and rehabilitation of Substance Use Disorders (SUDs), such as the therapeutic community adopted by the treatment facilities of the Coptic Church in Egypt serving both Muslim and Christian communities [4].

Substance Use Disorders are a group of chronic and relapsing disorders characterised by pathological and habitual drug seeking behaviours and compulsive drug use despite the negative consequences [5]. Current treatment approaches for SUDs are therefore complex and vary in modalities [6].

Egypt has taken steps since 1991 to combat drug addiction and abuse through the activities of the Fund for Drug Control and Treatment of Addiction (FDCTA). These services are communicated to the public through hotline and media campaigns. For example, a recent 24-h hotline initiative received a total of 81,777 calls seeking advice and support from people and families struggling with addiction and drug abuse. Furthermore, early detection tests have been carried out with 12,000 school bus drivers [7].

Treatments programs for SUDs in Egypt target motivation, using Cognitive Behavioural Therapy (CBT) and prevention strategies. The out-patient programs cover diagnosis, screening, motivational interviewing, provision of drugs for home detoxification, and follow-up. In-patient programs consist of detoxification phase and rehabilitation. Day care programs which follow the successful completion of rehabilitation phase and aim to maintain abstinence [8]. Additionally, a harm reduction model is implemented in Egypt for injecting drug users which includes anonymous peer education on safe sex and safe drug injection and medical services for sexually transmitted infections [9].

Recovery from SUDs involves change in more than substance use related outcomes, and can involve positive personality changes [10]. Recovery from SUDs can be defined as a personal change process which can be reached through treatments to achieve sobriety [11]. The Substance Abuse and Mental Health Services Administration (SAMHSA) defined recovery as positive change process through which individuals improve their health, wellness, and lead goal directed lives [12]. Recovery extends beyond abstinence from substance use, it involves developing sense of well-being and quality of life [13]. Achieving abstinence is a consensus outcome for recovery from SUDs [14].

Integrating peers, defined as people with lived experience of SUDs, into the delivery of care for people recovering from SUDs has shown favorable outcomes [15, 16]. Regardless of the nature of their role, peers have the capabilities to engage service users outside the limitations of traditional clinical practice [17]. SAMHSA defined peer support for SUDs as “a set of non-clinical, peer-based activities that engage, educate, and support individuals so that they can make life changes that are necessary to recovery from substance use disorders” [18]. Peer-based recovery support or recovery coaching is delivered through formal and structured roles, compared to mutual aid support which is provided through informal pathways and does not necessarily require formal training [19]. Fundamental principles exist to identify the key values of working as a Peer Support Worker (PSW): mutual support should exist between people who share not only the mental health condition but also similar life experiences; experiential learning as a journey; and encouraging natural social support [20]. Incorporating Peer Support Workers (PSWs) in the workforce enhances mental health system responsiveness to service users’ needs [21]. Favorable service user related outcomes are associated with the involvement of PSWs in the management of SUDs e.g. reduced relapse rates, and improved treatment retention and satisfaction with the treatment [22]. Regarding the impact of working as PSWs for SUDs on the PSWs themselves, the professional role has positively influenced PSWs’ own recovery and provided them with a sense of purpose. Validation of change, increased confidence, and improved finances were experienced by PSWs. Additionally, less stigma, more empowerment, positive sense of identity and enhanced social network through interaction with other fellow peer workers have also been reported [23–26].

Multifaceted organizational and workforce challenges facing PSWs for SUDs exist: lack of role clarity, poorly defined job description, boundary issues and low salaries [27, 28]. However, mental health systems have been assisting peers to play an integral role in service delivery, particularly regarding the provision of training and technical assistance [29]. Involvement of PSWs in the co-production and co-creation of mental health and addiction services by mental health systems were reported as one aspect of the PSWs role in the organization [30].

The majority of the published work on peer support for SUDs is mainly geared toward the effectiveness of the role for service users [31], however, the influences on integration of peers into the wider mental health system needs to be more understood [32]. In Egypt, peer support work for substance use disorders exists in both public and private mental health facilities. To our knowledge, no Egyptian studies have been published about peer support work.

### Aim

The aim of this study is to explore the experiences of peer support workers for substance use disorders in Egypt. Exploring lived experience is more than simply collecting facts, it is concerned with building upon experiential subjective knowing in real time and space with responsiveness to chronological events [33].

## Methods

### Design

Descriptive phenomenological [34] qualitative research design. Phenomenological research design is concerned with examining and reflecting on the lived experiences of research participants [35].

### Participants and recruitment

Participants were identified by means of purposive and snowball sampling through professional (formal) and personal (informal) networks and through social media announcements.

### Inclusion criteria

1. Adults (18–60 years old) who are employed as PSWs for SUDs, defined as those who received structured training to work as a formal PSW and are receiving a salary.
2. Had a former diagnosis of SUDs
3. PSWs in either private or public settings.

### Exclusion criteria

1. Unpaid formal or informal peer support workers and other recovery volunteers as the majority of them lack formal training
2. Individuals who did not receive structured training for PSW role

### Ethical considerations

Faculty of Nursing, Mansoura University, Egypt, Research Ethics Committee approval was obtained (Ref. No. P.0228) prior to participant recruitment. Verbal consent was obtained after circulating the study information sheet to participants electronically and before commencing with the interviews. Confidentiality, anonymity, the right to withdraw at any time during the study, and methods of handling recorded data were explained to participants.

### Procedure

A semi-structured topic guide was designed, including open ended questions about role definition, professional trainings, qualifications, experience of working with multidisciplinary teams, and organizational support for PSWs for SUDs. Interviews were conducted by the first author in vernacular Egyptian Arabic. Three pilot interviews were conducted to ensure the clarity of the questions and update the topic guide if needed. The pilot interviews were included in the analysis as no changes were applied to topic guide.

Participants were recruited through formal and informal networks of the authors and via social media platforms (Facebook and Twitter). Participants were interviewed via password protected video conferencing (Zoom) or through telephone conferencing in accordance to their preference and availability.

The average length of interviews was 50 min. Verbal consent was obtained and recorded. Recruitment and interviewing took place until data saturation was reached. All recorded interviews were transcribed and translated to English by DA and AS. To check the quality of translations, three transcripts were back translated to Arabic independently by the first author (NI), then all translations were reviewed by a professional English translator.

### Analysis

To achieve an understanding of the patterns of meaning in the data, thematic analysis based on descriptive phenomenology was followed. This approach differs from descriptive phenomenological approach in organizing pattern of meanings into themes which contribute to the robustness of findings. Thematic analysis based on descriptive phenomenology focuses on meanings in the data rather than measuring its frequency [36]. The primary analyst (NI) familiarized herself with the data by reading transcripts several times to explore experiences embedded in the data, meanings were emphasized holistically rather than presenting its frequency or count. The analyst moved back and forth between the whole and the parts of the data and used short descriptive words for each line to give meanings names. Meaning were then related to each other’s to develop a pattern. Data organization was performed by NVivo 12 software. A secondary analyst (AE) independently coded five interviews and met with the primary analyst for discussion to develop the coding framework. When agreement between the researchers were achieved on the coding framework, NI applied the coding framework to the remaining interviews.

## Results

### Participant characteristics

A total of 17 PSWs participated. All were men and their mean age was 41.5 ± 8.2 years. Participants came from different governorates in Egypt Cairo (*n* = 6), Dakahliya (*n* = 5), Alexandria (*n* = 3), Al-Minya (*n* = 2) and Al-Qalybiya (*n* = 1). Eight worked in private and nine in public facilities. The average duration of participants’ working as PSWs was five years. The sample included three PSW managers.

### Elicited themes

Three superordinate themes were identified: 1) Role responsibility 2) Relationship with colleagues and organization, and 3) Challenges to the role integrity. The coding framework is presented in Table 1.

**Table 1:** Coding framework for role of peer support workers’ experiences for substance use disorders in Egypt

Main theme	Examples
**Theme 1: Role clarity** *1.1 Building therapeutic relationships with service users and instillation of hope*	*“Addiction therapists (he is referring to PSWs) can make patients believe that there is always hope for recovery by telling them how they gave up on addiction and their personal recovery stories. Particularly when the patient is first admitted to the treatment center, he is in denial of his addiction problem or he thinks that his addiction cannot be treated, so therapist will represent hope for him” (≠ PSW 05)* *“At first, the addiction therapist (he is referring to PSW) should be a role model and a recovered addict before being a therapist, this fact connects the therapist directly to the patient and opens the door for therapist- patient relationship to exist. The patient first enters the treatment facility with his own judgements and trust issues, the idea of him putting his trust on an ex addict requires first seeing a model that can give him hope, and be his first step towards recovery, and before conducting any therapeutic technique, this should be the first thing to be considered from addiction therapists” (≠ PSW 16)* *“The therapist (he is refereeing to PSW) tries to be closer to the patient, especially when the patient knows that he is with someone who was in the same situation but recovered and stopped addiction, this gives the patient hope” (≠ PSW 17)* *“I use my personal experience as a kind of hope, when I like to convey hope; how I was able to recover and change, and where I am now” (≠ PSW 04)* *“I don't say all the time I have a lived experience, I let the patient see that we share commonalities” (≠ PSW 07)* “*You bring out the motivation from inside the patient, do not tell him what to do, I let him say his problems, and I ask him what he needs, he says I need to stop the addiction, he is the one who decides” (≠ PSW03)* *“I do not control the patient, I am not a police officer, and I always say that I am dealing with souls and human beings who need you to provide them with motivation and positive feelings” (≠ PSW13)* “*Motivational interviewing is important as a beginning step with the patient when he comes first to the treatment facility, the patient comes with denial and lack of motivation to change. Here it comes the role of the addiction therapist to motivate the patient to be engaged in treatment” (≠ PSW10)* *“Addition therapist (he is referring to PSWs) must have professional ethics, so that he can be responsible for the patient and gain this trust” (≠ PSW14)* *“The therapist (he is referring to PSW) should practice therapeutic limits in dealing with the patient, for example accepting gifts from patients is prohibited” (≠ PSW09)* *“In trainings, we study professional ethics academically, what is really important is to practice it in our professional career” (≠ PSW07)*
*1.2: Working with multidisciplinary teams*	*“Every successful addiction institution has addiction therapist (he is referring to PSW) and like everyone in the team, he has an important role in this process. unlike doctors, the addiction therapist is always available for the patient 24 h and has been through the same issue, so the team needs the doctor, nurse and the addiction therapist” (≠ PSW05)* *“We discuss different points of view regarding the treatment plan, but it is for the patient benefit. For example, yesterday there was a patient, we were concerned about giving him a holiday from the treatment facility, because the patient’s condition, he is not stable and has some problems, so we were consulting if he could take a break, because of the possibility that he might relapse. These are not difficulties, but they are judgments and points of view until we reach what is in the best of the patients at the end, and to do what is right. I advise him in the first place to be a part of the team, and not to consider himself as the patient’s healer alone” (≠ PSW01)* *“We have an integrated team; addiction therapist, addiction volunteer, doctor, and psychologist. We emphasize the psychological aspects behind SUDs, we perform psychological tests using psychometric questionnaires. We should understand the psychological reasons behind SUDs” (≠ PSW06.)* *“The decision is supposed to be collective because individual decisions are harmful, so when we all have an agreement on a specific point of view and my team members think this is not good for the current situation I don’t challenge and I do what we all agree on (≠ PSW02)* *“When I started my professional training, I learned that the therapeutic team consists of three pillars, an addiction therapist (he is referring to PSW), a psychiatrist, and a psychologist. I listen to the opinion of the doctor and the opinion of the specialist, we all discuss the case. There are places in Cairo that work in the family and marriage issues, few places that work in this way” (≠ PSW03)* *“If there is disagreement in the professional point of view, we vote. The treatment team includes a consultant, a doctor, a specialist, and a therapist, four or five people. When we disagree we vote” (≠ PSW12)*
*1.3: Professional training, peer-supervision, and self-care activities*	*“The addiction therapist (he is referring to PSWs) has his own recovery story but he must support it with professional training experience. The lived experience without professional training is not enough” (≠ PSW17)* *“I talk and share with supervisors, peer-meetings, it helps me a lot and help in re-charging my batteries” (≠ PSW11)* *“I personally like Narcotic Anonymous as a preparation program for being a professional addiction therapist and a rehabilitation and treatment program too” (≠ PSW08)* *“I attend peer group meetings regularly and talk about my problems and conflicts so I can be qualified to offer counseling and support to service users, because the therapist must keep working on resolving his problems that had not been solved during his recovery journey (≠ PSW01)* *“Since SUDs is a chronic disease and it continues with me for the rest of my life, I should keep managing it and work on my recovery because I can possibly relapse at any time. I share and participate in peer supervision meetings, I must also participate on personal aspects related to myself; I make mistakes, I have pressure on me and sometimes I have difficulty in managing my feelings, so I must always participate” (≠ PSW05)* *“The mentor is very important indeed, without the mentor we may relapse” (≠ PSW13)* *“It is very helpful to believe that there is someone who I can talk to and motivates me to continue, I am willing to always get back to him and discuss issues related to my work and personal recovery with. Supervision is really important” (≠ PSW10)* *I take a break and come back to work with a fresh mind, because I may have negative feelings that would affect patients negatively, and perhaps a word or a behaviour that come from me ruin the job with the patient if I am stressed” (≠ PSW04)* *“With the intense and hard responsibilities of this job, I always delegate duties, I have a deputy director and when I go for holidays, I completely turn my mind off” (≠ PSW06)*
*1.4: The therapeutic use of lived experience*	*“I use my story when the patient stops listening to us as a therapeutic team or when he gets into deep desperation, here it indicates that my consultation is not just coming from a professional place, so it is not necessary to use my experience all the time” (≠ PSW13)* *“You know what, for reassurance, sometimes I tell the patient that I have gone through addiction experience, so he can speak comfortably with me. However, there are smart patients who notice if the therapist talks about his experience a lot. The patient will feel that the therapist is only here to talk about himself and what he did in a bragging way. So one should be very careful” (≠ PSW14)* *“When we have an evasive or hard patient, we sometimes tell him that as addiction therapists we are like him, we share the same mind, our thinking is similar” (≠ PSW17)* *“When the client has negative thoughts and he can’t speak it up, here I can bring my personal experience to help him talk, and no need for many details, also in situations where he feels shameful to talk I encourage him by talking about me being the same. It all meant to make him not to feel alone” (≠ PSW08)*
*1.5: Knowledge and integration of therapeutic schools*	*“Besides formal training, addiction therapist should study different therapeutic schools, sometimes we use combination of therapeutic techniques from different schools with the patient” (≠ PSW16)* *“Generally, all therapeutic schools have things in common, they differ in the concepts, for example the Cognitive Behavioral Therapy (CBT) focuses on changing thinking, and the Narcotic Anonymous (NA) goes in steps and the patient must attend 90 meetings in 90 days with a supervisor attending with him, some programs have combined NA & CBT as the matrix model. I should first explain to the patient that all programs can work toward his recovery” (≠ PSW05)* *“Addiction therapist should be familiar with all therapeutic schools, he should not work in one way, one path may not succeed” (≠ PSW03)*
**Theme 2: PSWs’ need for organizational and stakeholders’ support**	*“Formal training to undertake a job as addiction therapist must be set mandatory by the policy makers, addiction therapists should be supported financially to undertake these training. That should be mandated by laws. Additionally, therapists should be evaluated and tested to ensure they are qualified enough to take this job role” (≠ PSW10)* *“You know that training courses are expensive, and it is not easily available too, when I was at the Al-Matar hospital, we used to find courses every two or three years, courses about the role of the addiction therapist and the role of the treatment counsellor. We take these courses at our own expense…silence, and you know that we have already lost a lot of money and financial resources during our recovery and treatment journey” (≠ PSW06)*
**Theme 3: Challenges to the role integrity**	*“The new types of abused drugs are challenging, because it has different presentation from the known substances. All the new types make the patient go through terrible long psychological withdrawal symptoms, and the patient’s condition is very unstable for a long time, he goes through a period of hope in five minutes, and five minutes feels like failure and frustration. The most difficult time is in the first month, he has many thoughts and his condition is unstable, he starts treatment and then goes in denial, and he wants to leave and does not want to complete the treatment. Stabilizing the case is the most challenging part in the treatment plan. The therapist needs to learn about these new drugs, this must be included in the professional training of addiction therapists.” (≠ PSW01)* *“Not everyone who has recovered from SUDs can work as an addiction therapist (he is referring to PSWs) and not anyone who has recovered and given up addiction for six or nine months can call himself an addiction therapist, we also have no support or union” (≠ PSW13)* *“Our media only shows the darker side of the addiction treatment facilities, and this is a catastrophic thing” (≠ PSW17)* *“Long shifts…. (deep sigh) … the addiction therapist works usually for four consecutive days which is a big burden, in the last day he may reach the limit and gets tired. He is an ex-addict and that must be considered and not forgotten “(≠ PSW09)* *“The parents have a very important role in the treatment Plan, however, we face big difficulties arising basically from low family awareness about recovery of SUDs, for example when we talk about a person who started treatment and, the parents begin to see him getting better, he eats and sleeps well, his mother says that she wants him to marry, he is now starting to recover and stand on his feet, he has to take responsibility, their thinking revolves around that they want him to settle down even if he is not ready for that step “(≠ PSW16)* *“Some private facilities do not separate between the financial issues with patients’ family and patients’ care. for example, a patient may be suitable to go outside the facility for a break or a holiday but the facility won’t allow this because of the debts family owe to the center, then the patient comes to me wondering why he couldn’t go out” (≠ PSW15)* *“Unfortunately, addiction training courses are optional, this is a catastrophic thing. For example, you can study eighteen months in an addiction treatment school and eventually the certificate is not accredited in Egypt, but it is recognized and accredited internationally” (≠ PSW12)*

#### Theme 1: role responsibility

Five main duties were described by participants: building therapeutic relationships with service users and instillation of hope; working with multidisciplinary teams; professional training, peer-supervision, and self-care activities; the therapeutic use of lived experience; and PSWs’ need for organizational and stakeholders support.

- 1.1: Building therapeutic relationships with service users and instillation of hope

Participants described how PSWs can establish therapeutic relationship with service users in their recovery from SUDs. Instilling hope and being a role model to service users was described by some participants, particularly through sharing their own recovery journey. Additionally, PSWs described how motivating service users in their recovery journey as a way of connecting and building therapeutic relationship with them. Practicing professional ethics and setting boundaries with service users were described by PSWs as a means for building professional relationships.

“I convey the message about how I started and how I got to where I am now and what I did. The client needs someone to make him believe that recovery from SUDs happens, the client can see this in the addiction therapist, then he will believe that there is recovery and the change is possible. The therapist must be a living example so the client aspires to become like him and reaches out what the therapist achieved. I have met many clients telling me that they hope to become like me and work in the same field as addiction therapists. If the client did not see this in the addiction therapist (he is referring to PSW), the situation might be different and his belief in recovery would be compromised” (≠PSW 06).

“I give them tasks to do and I support them so their spirits rise and they start to feel that they are being better persons. I reward them for everything positive they do. When I see them progressing and working hard on themselves, I give them a chance to be the group leaders, all of this make us close to each other’s” (≠ PSW 01).

*“*Addiction therapists (he is referring to PSWs) can make clients believe that there is always hope for recovery by telling them how they gave up on addiction and their personal recovery stories. Particularly when the client is first admitted to the treatment center, he is in denial of his addiction problem or he thinks that his addiction cannot be treated, so therapist will represent hope for him” (≠ PSW 05).

“At first, the addiction therapist (he is referring to PSW) should be a role model and a recovered addict before being a therapist, this fact connects the therapist directly to the client and opens the door for therapist- service user relationship to exist. The client first enters the treatment facility with his own judgements and trust issues, the idea of him putting his trust on an ex addict requires first seeing a model that can give him hope, and be his first step towards recovery, and before conducting any therapeutic technique, this should be the first thing to be considered from addiction therapists” (≠ PSW 16).

*“*Addiction therapists (he is referring to PSWs) can make clients believe that there is always hope for recovery by telling them how they gave up on addiction and their personal recovery stories. Particularly when the client is first admitted to the treatment center, he is in denial of his addiction problem or he thinks that his addiction cannot be treated, so therapist will represent hope for him” (≠ PSW 05).

“At first, the addiction therapist (he is referring to PSW) should be a role model and a recovered addict before being a therapist, this fact connects the therapist directly to the client and opens the door for therapist- relationship to exist. The client first enters the treatment facility with his own judgements and trust issues, the idea of him putting his trust on an ex addict requires first seeing a model that can give him hope, and be his first step towards recovery, and before conducting any therapeutic technique, this should be the first thing to be considered from addiction therapists” (≠ PSW 16)

“The therapist (he is refereeing to PSW) tries to be closer to the client, especially when the client knows that he is with someone who was in the same situation but recovered and stopped addiction, this gives the client hope” (≠ PSW 17).

“I use my personal experience as a kind of hope, when I like to convey hope; how I was able to recover and change, and where I am now” (≠ PSW 04).

“I don't say all the time I have a lived experience, I let the client see that we share commonalities” (≠ PSW 07).

“You bring out the motivation from inside the client, do not tell him what to do, I let him say his problems, and I ask him what he needs, he says I need to stop the addiction, he is the one who decides” (≠ PSW03).

“I do not control the service user, and I always say that I am dealing with souls and human beings who need you to provide them with motivation and positive feelings” (≠ PSW13).

“Motivational interviewing is important as a beginning step with the service user when he comes first to the treatment facility, the client comes with denial and lack of motivation to change. Here it comes the role of the addiction therapist to motivate the client to be engaged in treatment” (≠ PSW10).

“Addition therapist (he is referring to PSWs) must have professional ethics, so that he can be responsible for the client and gain this trust” (≠ PSW14)

“The therapist (he is referring to PSW) should practice therapeutic limits in dealing with the client, for example accepting gifts from clients is prohibited” (≠ PSW09).

“In trainings, we study professional ethics academically, what is really important is to practice it in our professional career” (≠PSW07).

- 1.2: Working with multidisciplinary teams

Participants reported that integrating and successfully navigating working with multidisciplinary members of the mental health team is one of the roles of the PSWs of SUDs. Learning how to discuss and solve disagreements and sharing decisions about service users’ treatment plan underpin PSW success in working with multidisciplinary teams.

“I am part of the treatment team, we share all things and decisions, for example a service user we want to decide a visit or a telephone call for him, and since I am the one who spends the most time with the client, I am consulted and asked if the service user is ready at the time being, this is important. each member of the team has his unique role” (≠PSW 09).

*“*Every successful addiction institution has addiction therapist (he is referring to PSW) and like everyone in the team, he has an important role in this process. unlike doctors, the addiction therapist is always available for the client 24 hours and has been through the same issue, so the team needs the doctor, nurse and the addiction therapist” (≠ PSW05).

“We discuss different points of view regarding the treatment plan, but it is for the client benefit. For example, yesterday there was a service user, we were concerned about giving him a holiday from the treatment facility, because the client’s condition, he is not stable and has some problems, so we were consulting if he could take a break, because of the possibility that he might relapse. These are not difficulties, but they are judgments and points of view until we reach what is in the best of clients at the end, and to do what is right. I advise him in the first place to be a part of the team, and not to consider himself as the client’s healer alone” (≠ PSW01).

“We have an integrated team; addiction therapist, addiction volunteer, doctor, and psychologist. We emphasize the psychological aspects behind SUDs, we perform psychological tests using psychometric questionnaires. We should understand the psychological reasons behind SUDs” (≠PSW06.).

“The decision is supposed to be collective because individual decisions are harmful, so when we all have an agreement on a specific point of view and my team members think this is not good for the current situation I don’t challenge and I do what we all agree on (≠PSW02).

“When I started my professional training, I learned that the therapeutic team consists of three pillars, an addiction therapist (he is referring to PSW), a psychiatrist, and a psychologist. I listen to the opinion of the doctor and the opinion of the specialist; we all discuss the case. There are places in Cairo that work in the family and marriage issues, few places that work in this way” (≠PSW03).

“If there is disagreement in the professional point of view, we vote. The treatment team includes a consultant, a doctor, a specialist, and a therapist, four or five people. When we disagree we vote” (≠PSW12).

- 1.3: Professional training, peer-supervision, and self-care activities

Peer support workers described that undertaking certain tasks is essential to function successfully. Participants described that their lived experience and their personal recovery only are insufficient for PSW practice. Additionally, professional training and continuing professional development activities are prerequisites to the role. Peer supervision was described by participants as a tool for maintaining their own personal recovery from SUDs and preventing relapse. Additionally, Self-care activities were described by PSWs with particular reference to the tough and demanding nature of working as PSWs for SUDs.

“What is currently available and reliable is “Freedom” diploma, and there are many courses for education. Recently I started to meet with a psychology professor to learn the psychological aspects behind SUDs. He teaches courses to PSWs for SUDs groups to learn emotional regulation strategies for SUDs” (≠PSW 04).

“I think about what do I want to talk about with someone like a supervisor or a senior colleague about my conflicts, I’m a person with a previous addictive behavior and I should always remember that conflicts may cause me a crisis” (≠PSW 02).

“I regularly take vacations and breaks, there are a lot of work pressures and other life pressures” (≠PSW 11).

*“*The addiction therapist (he is referring to PSWs) has his own recovery story but he must support it with professional training experience. The lived experience without professional training is not enough” (≠PSW17).

“I talk and share with supervisors, peer-meetings, it helps me a lot and help in re-charging my batteries” (≠PSW11).

“I personally like Narcotic Anonymous as a preparation program for being a professional addiction therapist and a rehabilitation and treatment program too” (≠PSW08).

“I attend peer group meetings regularly and talk about my problems and conflicts so I can be qualified to offer counseling and support to service users, because the therapist must keep working on resolving his problems that had not been solved during his recovery journey (≠PSW01).

“Since SUDs is a chronic disease and it continues with me for the rest of my life, I should keep managing it and work on my recovery because I can possibly relapse at any time. I share and participate in peer supervision meetings, I must also participate on personal aspects related to myself; I make mistakes, I have pressure on me and sometimes I have difficulty in managing my feelings, so I must always participate” (≠PSW05).

“The mentor is very important indeed; without the mentor we may relapse” (≠PSW13).

“It is very helpful to believe that there is someone who I can talk to and motivates me to continue, I am willing to always get back to him and discuss issues related to my work and personal recovery with. Supervision is really important” (≠PSW10).

I take a break and come back to work with a fresh mind, because I may have negative feelings that would affect service users negatively, and perhaps a word or a behaviour that come from me ruin the job with the client if I am stressed” (≠PSW04).

“With the intense and hard responsibilities of this job, I always delegate duties, I have a deputy director and when I go for holidays, I completely turn my mind off” (≠PSW06).

- 1.4: The therapeutic use of lived experience

Lived experience was described as one of the strongest therapeutic tools PSWs of SUDs possess. Participants reported that their lived experience is a unique quality that differentiates them from anyone in the mental health team. Additionally, PSWs described some processes used when disclosing their own story with service users:

“So, I know exactly what is it like to be there, and when he knows that I had been using for a longer period and sometimes stronger drugs than he does, the client will start to ask me about my journey. I had been sitting in the same chair” (≠ PSW 04).

*“*I use my story when the client stops listening to us as a therapeutic team or when he gets into deep desperation, here it indicates that my consultation is not just coming from a professional place, so it is not necessary to use my experience all the time” (≠PSW13).

“You know what, for reassurance, sometimes I tell the client that I have gone through addiction experience, so he can speak comfortably with me. However, there are smart clients who notice if the therapist talks about his experience a lot. The client will feel that the therapist is only here to talk about himself and what he did in a bragging way. So one should be very careful” (≠PSW14).

“When we have an evasive or hard client, we sometimes tell him that as addiction therapists we are like him, we share the same mind, our thinking is similar” (≠PSW17).

“When the client has negative thoughts and he can’t speak it up, here I can bring my personal experience to help him talk, and no need for many details, also in situations where he feels shameful to talk, I encourage him by talking about me being the same. It all meant to make him not to feel alone” (≠PSW08).

- 1.5: Knowledge and integration of therapeutic schools

PSWs emphasized that knowledge and integration of different therapeutic schools is an essential part of the PSW role.

“The therapist (he is referring to PSW) should have knowledge of different schools, because sometimes choosing the school depends on client’s condition, sometimes the client needs integration of different schools, according to what the client needs, but I prefer to start with Cognitive Behavioural Therapy (CBT); as they always come in denial and altered thoughts” (≠PSW 11).

*“*Besides formal training, addiction therapist should study different therapeutic schools, sometimes we use combination of therapeutic techniques from different schools with the client” (≠PSW16).

“Generally, all therapeutic schools have things in common, they differ in the concepts, for example the Cognitive Behavioral Therapy (CBT) focuses on changing thinking, and the Narcotic Anonymous (NA) goes in steps and the client must attend 90 meetings in 90 days with a supervisor attending with him, some programs have combined NA & CBT as the matrix model. I should first explain to the client that all programs can work toward his recovery” (≠PSW05).

“Addiction therapist (refereeing to PSW) should be familiar with all therapeutic schools, he should not work in one way, one path may not succeed” (≠PSW03).

#### Theme 2: PSWs’ need for organizational and stakeholders support

Participants stressed their need for stakeholders’ support (e.g. Ministry of Health and Population and the General Secretariat of Mental Health in Egypt) to undertake training courses to function as PSWs. Participants emphasized that these training courses should be mandatory for people with lived experience who have recovered from SUDs and interested in working as PSWs.

“Stakeholders should also give attention to all addiction therapists (he is referring to PSWs) and help them to develop themselves by launching programs and training courses for them. The mental health facility should trust their professional opinions and judgements when it comes to client’s decisions. You know if every governorate in our country supervised all addiction therapists and evaluate their suitability for formal roles, that will easily unlock their potentials and they can add high value to the clients” (≠PSW 14).

“You know that training courses are expensive, and it is not easily available too, when I was at the “Al-Matar” hospital, we used to find courses every two or three years, courses about the role of the addiction therapist and the role of the treatment counsellor. We take these courses at our own expense…silence, and you know that we have already lost a lot of money and financial resources during our recovery and treatment journey” (≠PSW06).

*“*Formal training to undertake a job as addiction therapist (he is referring to PSW) must be set mandatory by the policy makers, addiction therapists should be supported financially to undertake these training. That should be mandated by laws. Additionally, therapists should be evaluated and tested to ensure they are qualified enough to take this job role” (≠PSW10).

#### Theme 3: challenges to the role integrity

Lack of role definition, lack of governmental support, the tough nature of the job, and the stigma toward PSWs were expressed by participants in the current study.

“There is a big confusion about the role definition of addiction therapists (referring to PSWs), every job has a job description that explains its role. The right question is when a person should be called an addiction therapist? The job is not even recognized in the country's registry for jobs’ records, for example, it is not possible to write on my National ID card that I am an addiction therapist (he is referring to PSW)” (≠PSW 15).

“Our media only shows the darker side of the addiction treatment facilities, and this is a catastrophic thing” (≠PSW17).

*“*The new types of abused drugs are challenging, because it has different presentation from the known substances. All the new types make the client go through terrible long psychological withdrawal symptoms, and the client’s condition is very unstable for a long time, he goes through a period of hope in five minutes, and five minutes feels like failure and frustration. The most difficult time is in the first month, he has many thoughts and his condition is unstable, he starts treatment and then goes in denial, and he wants to leave and does not want to complete the treatment. Stabilizing the case is the most challenging part in the treatment plan. The therapist needs to learn about these new drugs, this must be included in the professional training of addiction therapists.” (≠PSW01).

“Not everyone who has recovered from SUDs can work as an addiction therapist (he is referring to PSWs) and not anyone who has recovered and given up addiction for six or nine months can call himself an addiction therapist, we also have no support or union” (≠PSW13).

“Our media only shows the darker side of the addiction treatment facilities, and this is a catastrophic thing” (≠PSW17).

“Long shifts…. (deep sigh) … the addiction therapist (he is referring to PSW) works usually for four consecutive days which is a big burden, in the last day he may reach the limit and gets tired. He is an ex-addict and that must be considered and not forgotten “(≠PSW09).

“The parents have a very important role in the treatment Plan, however, we face big difficulties arising basically from low family awareness about recovery of SUDs, for example when we talk about a person who started treatment and, the parents begin to see him getting better, he eats and sleeps well, his mother says that she wants him to marry, he is now starting to recover and stand on his feet, he has to take responsibility, their thinking revolves around that they want him to settle down even if he is not ready for that step “(≠PSW16).

“Some private facilities do not separate between the financial issues with client’s family and clients care. for example, a client may be suitable to go outside the facility for a break or a holiday but the facility won’t allow this because of the debts family owe to the center, then the client comes to me wondering why he couldn’t go out” (≠PSW15).

“Unfortunately, addiction training courses are optional, this is a catastrophic thing. For example, you can study eighteen months in an addiction treatment school and eventually the certificate is not accredited in Egypt, but it is recognized and accredited internationally” (≠PSW12).

## Discussion

The current study aimed to explore the in-depth role of PSWs in the recovery of people with SUDs in Egypt. To our knowledge, no studies have been conducted in Egypt about peer support work for SUDs. The key findings were an articulation of five components of the PSW role, an identification of the importance of support from the hosting organization and from colleagues, and an exploration of Egypt-specific issues for PSWs.

There is no structured governmental certification procedure or training policy for PSWs for SUDs in Egypt. PSWs usually have different job titles such as recovery coach or addiction therapist, not peer support worker. This may reflect the stigma attached to PSW; some participants in the current study mentioned that they sometimes are treated as an “addict” by non-peer staff. People with Substance Use Disorders (SUDs) in Egypt struggle with stigmatizing social labels placed on them [37]. The majority of PSWs for SUDs in Egypt informally undergo the twelve steps program of Narcotic Anonymous (NA) [38], the Matrix model [39], or Cognitive Behavioural Therapy (CBT) training [40] to work as professional PSWs. People with lived experience of recovery from substance use disorders who did not receive any formal training can work as supervisors who help service users who closely monitor service users during their rehabilitation program and provide custodial care to them at recovery houses to achieve recovery goals. One well-known Christian private facility provides a training diploma (International Substance Abuse & Addiction Coalition, ISAAC) in collaboration with the NET Institute (Center for Addiction and Recovery Education https://netinstitute.org) in the United States to prepare PSWs for SUDs to undertake a professional role. This training diploma is prestigious and its graduates are recognised in the field in Egypt as it follows the international standards of certification and training. This differs to mental health peer support models in high-income countries/Western countries [41] where PSWs have access to training in using their lived experience with others, however are not required to undergo formal training in a therapeutic orientation (e.g. CBT). In Egypt, mutual giving and receiving, natural support and reciprocity exist where PSWs share and explore with their peers, which is an essential aspect of their role. This extends beyond sharing the lived experience between PSWs and service users. However, no references about that exist so far, as this area is still under-researched in Egypt.

According to the systematic review by Bassuk and colleagues, inconsistencies exist in the studies concerning the definition of peer workers and recovery coaches. Additionally, most studies lacked a clear description of their roles and responsibilities. The lack of consistency was attributed to the wide range of existing training programs [19]. Research is needed to delineate the type of support PSWs give and the type of professional supervision they undergo [42]. 

The current study found the PSW role comprises five main responsibilities. First, building therapeutic relationship with service users during their recovery from SUDs. Participants described how instilling hope and acting as a role model to service users enhances building the relationship with the service users. A fundamental aspect of peer relationship is conveying the message of hope through sharing lived experience that makes service user feel that recovery from SUDs is sustainable [44, 45]. In a study by Pauly and Mamdani [46], PSWs reported that being an inspiration for service users for SUDs is among the motivators for PSWs to continue in the job. Another factor that supported the building of a therapeutic relationship with service users which was reported by participants in the current study was motivating service users during their recovery journey. This motivational element corresponds with findings of Gressler and colleagues which revealed that motivating service users during initiation and maintenance of treatment support the therapeutic relationship between the service user and PSW [47]. Another aspect contributing to building therapeutic relationship with service users as described by PSWs is practicing professional ethics and maintaining boundaries with service users. Only three participants in the current study contributed to this finding, which may be related to the lack of professional code of ethics for PSWs which warrants attention for future training for PSWs.

Second, participants reported working with multidisciplinary teams as the second duty of PSWs of SUDs, even though the medical model still dominates mental health practice in Egypt [48]. Voting and discussions between mental health team members were described by participants as tools for resolving disagreements in decisions. This may be attributed to the understanding of mental health team members that PSWs possess the unique attribute of their lived experience that makes a distinctive contribution to team decision-making. This corresponds with results of Ehrlich and colleagues who reported that PSWs were successful in navigating among the interprofessional mental health team, particularly when teams focus on the unique qualities PSWs bring [49].

The third role described by PSWs is professional training, peer-supervision, and self-care activities. Although no formal training policy exists for PSWs for SUDs, PSWs believe that the lived experience only is not enough to lead a professional role as a PSW. Participants described the high cost and limited access to PSW training, particularly with low pay and long working shifts of PSWs in Egypt, so not everyone can access it. Different training approaches employed in Egypt for PSWs warrants attention as essential elements in PSW training may be missing and a consensus should be reached to establish essential training components as the case in the Delphi study of Charles and colleagues [43]. Organizations which assist PSWs to combine the technical and competency-based aspect of the job through training with the reflexive, value-driven, and artistic aspect of PSW (lived experience) will enhance the professionalism of PSWs [50].

Peer supervision was described as an essential activity for professional PSWs for SUDs. All participants in the current study described how peer supervision maintains their own recovery and protect them from relapse which is consistent with findings of Chapman and colleagues where one-to-one or group supervision of PSWs was highlighted as a supportive factor to the recovery of PSWs themselves and help in addressing boundaries issues PSWs may go through with service users [51]. However, according to Phillips [52], PSW supervisors help peer workers to remain grounded within the scope of peer support roles. According to Price [53], transformational leadership happens as an idealized influence occurs through communication between PSWs and their supervisors; PSWs perceive their supervisors as a role model who enable them address the challenges they face in clinical practice. According to Phillips and Harrison [54], supervision of PSWs should focus on reflective practice, helping in setting professional goals, boundaries and professional ethics, and work-place wellness strategies.

Among the activities PSWs described in the current study are self-care and wellbeing activities particularly due to the intense nature of working as PSWs of SUDs. Self-care activities have been associated with promoting well-being in the workplace, enhancing work/life balance, and decreasing the potential for emotional burnout [55].

The fourth role described by participants was using lived experience as a therapeutic tool with the service users. Participants’ views varied regarding when to use their lived experience with service users. Participants reported using their personal experience as a tool to build therapeutic relationship with service users and to instill hope when they feel hopeless or ashamed. According to the systematic review of Satinsky and colleagues [56] using lived experience enhances engagement of service users in treatment in low-middle income countries. Lived experience may combat the stigma around PSW for SUDs in Egypt, however PSWs may be at higher risks than other non-peer professionals of personalizing service users’ success and failures which was attributed to the use of lived experience. Additionally, PSWs using their lived experience in supporting recovery of people with SUDs may place peer workers at higher risks for relapse. Ineffective ability to use lived experience may negatively impact personal recovery of PSWs and their well-being [57–59].

The second superordinate theme elicited was relationship with colleagues and organization. A need for a role support through governmental training and certification policy was expressed by PSWs. A systematic review published by Ibrahim and colleagues identified that PSW training is among the facilitators of implementing PSW in mental health settings [60].

The final superordinate theme was challenges to the role integrity, including lack of accredited training and certification programs for PSWs in Egypt and the stigma facing PSWs in Egypt. In Egypt substance use is usually associated with negative stereotypes from both the public and health care professionals [61]. In the current study some PSWs reported stigmatizing attitude from their non-peer co-workers, consistent with Walker and Bryant who reported that PSWs revealed experiences of prejudice and discrimination from non-peer colleagues at work [62].

The results of the current study can inform mental health policy in Egypt, particularly promoting organizational support policies and informing the design of accreditation and certification programs for PSWs of SUDs. The findings indicate a need for national and governmental support to PSWs of SUDs in both public and private facilities, along with consensus about role definition and consideration of certification and accreditation programs for PSWs. Organizational culture training is needed for non-peer workers to enhance their understanding of the role of PSWs in the treatment of SUDs. Stigma-reducing interventions are also needed to support the disclosure of lived experience, and the media can help in changing the negative stereotypes about people with substance use disorders and PSWs. Further research about PSW in an Egyptian context is warranted, including consideration of investigating female PSWs role both in Egypt and globally.

### Strengths and limitations

This is the first study to explore the role of PSWs of SUDs in Egypt. Strengths include the recruitment of PSWs from both public and private facilities, hence increasing the credibility of findings across different setting. Although the inclusion criteria of the current study included both male and female PSWs for SUDs, all participants were men. Female PSWs for SUDs in Egypt do exist and were approached to participate, but perhaps because of stigmatizing feelings, they either did not respond or declined participation in the study. According to Watters and Biernacki, some population or research participants are considered invisible, hidden, or hard to reach; among them are women who struggle with SUDs [63]. During drug use and recovery, women with SUDs experience more stigma, shame, less social support, and issues with their parental and custody roles compared to their male counterparts. They require gender responsive efforts in the design and recruitment phase of research [64]. Additionally, non-involvement of PSWs in the design of the topic guide is considered a limitation of this study. Finally, the available data only allowed preliminary exploration of the third superordinate theme of Egypt-specific challenges, and future work might explore these issues, such as the impact of cultural values and stigma on PSW practice. Formal evaluation of peer support work is underway in other low and middle income countries [65, 66].

## Data Availability

The datasets generated and/or analysed during the current study are not publicly available as it contains recorded interviews and personal information which participants did not consent to being released.

## References

[1] Rabie M, Shaker NM, Gaber E, El-Habiby M, Ismail D, El-Gaafary M, Lotfy A, Sabry N, Khafagy W, Muscat R (2020). Prevalence updates of substance use among Egyptian adolescents. Middle East Curr Psychiatr.

[2] Middle East and North Africa Harm Reduction Association (MENAHRA). Situation on Assessment of Situation and Response of Drug Use and its Harms in the Middle East and North Africa 2020; 2021. Retrieved from http://www.menahra.org/images/pdf/Situation_Assessment_2021_-_Web.pdf

[3] Ahmed Y, Ramadan R, Sakr MF. Equity of health-care financing: a progressivity analysis for Egypt. J Humanit Applied Soc Sci. 2020. Retrieved from 10.1108/JHASS-08-2019-0040/full/html

[4] UNODC (2004). Strengthening The Treatment And Rehabilitation Services For Drug Abusers In Egypt And Jordan.

[5] Vandaele Y, Janak PH (2018). Defining the place of habit in substance use disorders. Prog Neuropsychopharmacol Biol Psychiatry.

[6] Monteiro MG (2002). The Evaluation of Treatment for Substance Use Disorders: Relevance to.

[7] National efforts in comating drugs (on the occasion of the international days of combating drug abuse and illicit trafficking). 2020. International Human Rights Days Reports. Supreme Standing Committee for Human Rights. Retrieved from hrd-5-2020-en.pdf (sschr.gov.eg)

[8] Saeed SIN. Aims of Egypt: Assessment of Governmental Mental Health System Egypt (2016-2017) (Doctoral dissertation). 2018. Retrieved from https://run.unl.pt/handle/10362/40174

[9] Oraby D (2013). Harm reduction approach in Egypt: the insight of injecting drug users. Harm Reduct J.

[10] Ashford RD, Brown A, Brown T, Callis J, Cleveland HH, Eisenhart E, Groover H, Hayes N, Johnston T, Kimball T, Manteuffel B (2019). Defining and operationalizing the phenomena of recovery: a working definition from the recovery science research collaborative. Addict Res Theor.

[11] Yanushka TP. The Lived Experience of Recovery from Substance Use Disorder by Ex-offenders at a Faith-based Recovery Residence (Doctoral dissertation, Capella University).

[12] Yarbrough K. Religious and Spiritual Facets Influencing Substance Use Disorder Recovery (Doctoral dissertation, Alliant International University)..

[13] Kelly JF, Greene MC, Bergman BG (2018). Beyond abstinence changes in indices of quality of life with time in recovery in a nationally representative sample of US adults. Alcohol Clin Exp Res..

[14] Scott CK, Foss MA, Dennis ML (2005). Pathways in the relapse—treatment—recovery cycle over 3 years. J Subst Abuse Treat.

[15] Englander H, Gregg J, Gullickson J, Cochran-Dumas O, Colasurdo C, Alla J, Collins D, Nicolaidis C (2020). Recommendations for integrating peer mentors in hospital-based addiction care. Subs Abus.

[16] Bassuk EL, Hanson J, Greene RN, Richard M, Laudet A (2016). Peer-delivered recovery support services for addictions in the United States: a systematic review. J Subst Abus Treat.

[17] Eddie D, Hoffman L, Vilsaint C, Abry A, Bergman B, Hoeppner B, Weinstein C, Kelly JF (2019). Lived experience in new models of care for substance use disorder: a systematic review of peer recovery support services and recovery coaching. Front Psychol.

[18] Reif S, Braude L, Lyman DR, Dougherty RH, Daniels AS, Ghose SS, Salim O, Delphin-Rittmon ME (2014). Peer recovery support for individuals with substance use disorders: assessing the evidence. Psychiatr Serv.

[19] Bassuk EL, Hanson J, Greene RN, Richard M, Laudet A (2016). Peer-delivered recovery support services for addictions in the United States: a systematic review. J Subst.

[20] Murphy R, Higgins A. The complex terrain of peer support in mental health: What does it all mean?. J Psychiatr Ment Health Nurs. 2018;25(7):441–8. 10.1111/jpm.1247424. 10.1111/jpm.12474 29797631

[21] Aakerblom KB, Ness O (2021). Peer support workers in co-production and co-creation in public mental health and addiction services: protocol for a scoping review. PLoS ONE.

[22] Eddie D, Hoffman L, Vilsaint C, Abry A, Bergman B, Hoeppner B, Weinstein C, Kelly JF (2019). Lived experience in new models of care for substance use disorder: a systematic review of peer recovery support services and recovery coaching. Front Psychol.

[23] Scannell C (2021). By helping others we help ourselves: insights from peer support workers in substance use recovery. Adv Ment Health.

[24] Moak VL. Case Study of Lived Experiences: Three Male Peer Recovery Coaches at a Community-based, Spiritual, Residential Substance Abuse Recovery Program. 2022. Retrieved from https://digitalcommons.liberty.edu/doctoral/3356/

[25] Trachtenberg M, Parsonage M, Shepherd G, Boardman J (2013). Peer support in mental health care: is it good value for money?.

[26] Mancini MA, Hardiman ER, Lawson HA (2005). Making sense of it all: consumer providers' theories about factors facilitating and impeding recovery from psychiatric disabilities. Psychiatr Rehabil J.

[27] Otte I, Werning A, Nossek A, Vollmann J, Juckel G, Gather J (2020). Challenges faced by peer support workers during the integration into hospital-based mental health-care teams: results from a qualitative interview study. Int J Soc Psychiatry.

[28] Miyamoto Y, Sono T (2012). Lessons from peer support among individuals with mental health difficulties: a review of the literature. Clin Pract Epidemiol Ment Health.

[29] Myrick K, Del Vecchio P (2016). Peer support services in the behavioral healthcare workforce: state of the field. Psychiatr Rehabil J.

[30] Aakerblom KB, Ness O (2021). Peer support workers in co-production and co-creation in public mental health and addiction services: Protocol for a scoping review. PLoS ONE.

[31] Scannell C (2020). Exploring the Dual Role of Consumer and Provider in Substance Use Peer Support Workers [D.Psy.].

[32] Scannell C (2021). Voices of hope: substance use peer support in a system of care. Subst Abus-Res Treat.

[33] Schwandt TA, Burgon H (2006). Evaluation and the study of lived experience.

[34] Jackson C, Vaughan DR, Brown L (2018). Discovering lived experiences through descriptive phenomenology. Int J Contemp Hosp Manag.

[35] Alase A (2017). The interpretative phenomenological analysis (IPA): a guide to a good qualitative research approach. Int J Educ Literacy Stud.

[36] Sundler AJ, Lindberg E, Nilsson C, Palmér L (2019). Qualitative thematic analysis based on descriptive phenomenology. Nurs Open.

[37] Gutierrez A (2015). The discourse of drug use in Egypt: an interdisciplinary exploratory study.

[38] Nash AJ (2020). The twelve steps and adolescent recovery: A concise review. Subst Abus.

[39] EhteshamiPouya S, Momtazi S, Makri A, Eskandari Z, Dadashi M (2018). The efficacy of matrix model treatment in the reduction of addiction severity and relapse prevention among amphetamine abusers. J Adv Med Biomed Res.

[40] Kouimtsidis C, Reynolds M, Drummond C, Davis P, Tarrier N (2007). Cognitive-behavioural therapy in the treatment of addiction.

[41] Livingston JD, Milne T, Fang ML, Amari E (2012). The effectiveness of interventions for reducing stigma related to substance use disorders: a systematic review. Addiction.

[42] Hymes AS (2015). A phenomenological study of the experiences of substance abuse peer recovery coaches career motivation and professional experiences (Doctoral dissertation, The University of North Carolina at Charlotte).

[43] Charles A, Nixdorf R, Ibrahim N, Meir LG, Mpango RS, Ngakongwa F, Nudds H, Pathare S, Ryan G, Repper J, Wharrad H (2021). Initial training for mental health peer support workers: Systematized review and international Delphi consultation. JMIR Mental Health.

[44] Boisvert RA, Martin LM, Grosek M, Clarie AJ (2008). Effectiveness of a peer-support community in addiction recovery: participation as intervention. Occup Ther Int.

[45] Davidson L, White W, Sells D, Schmutte T, O'Connell M, Bellamy C, Rowe M (2010). Enabling or engaging? the role of recovery support services in addiction recovery. Alcohol Treat Q.

[46] Pauly BB, Mamdani Z, Mesley L, McKenzie S, Cameron F, Edwards D, Howell A, Knott M, Scott T, Seguin R, Greer AM (2021). “It's an emotional roller coaster… But sometimes it's fucking awesome”: meaning and motivation of work for peers in overdose response environments in British Columbia. Int J Drug Policy.

[47] Gressler LE, Natafgi NM, DeForge B, Shaneman-Robinson B, Welsh C, Shaya F (2019). What motivates people with substance use disorders to pursue treatment? a patient-centered approach to understanding patient experiences and patient-provider interactions. J Subst Use.

[48] Ibrahim N, Ghallab E, Ng F, Eweida R, Slade M (2022). Perspectives on mental health recovery from Egyptian mental health professionals: a qualitative study. J Psychiatr Ment Health Nurs.

[49] Ehrlich C, Slattery M, Vilic G, Chester P, and Crompton D. What happens when peer support workers are introduced as members of community-based clinical mental health service delivery teams: a qualitative study. J Interprof Care. 2019. 10.1080/13561820.2019.1612334 10.1080/13561820.2019.1612334 31106671

[50] Walker G, Bryant W (2013). Peer support in adult mental health services: a metasynthesis of qualitative findings. Psychiatr Rehabil J.

[51] Chapman SA, Blash LK, Mayer K, Spetz J (2018). Emerging roles for peer providers in mental health and substance use disorders. Am J Prev Med.

[52] Price IM. Best Practices for the Supervision and Organizational Support of Peer Support Specialists (Doctoral dissertation, Argosy University, Atlanta). Retrieved from https://www.proquest.com/docview/2531017147?pq-origsite=gscholar&fromopenview=true

[53] Price TL. The ethics of authentic transformational leadership. The leadership quarterly. 2003;14(1):67–81.

[54] Phillips K, Harrison J (2019). Supervising Peer Workers.

[55] Bressi SK, Vaden ER (2017). Reconsidering self care. Clin Soc Work J.

[56] Satinsky EN, Kleinman MB, Tralka HM, Jack HE, Myers B, Magidson JF (2021). Peer-delivered services for substance use in low-and middle-income countries: a systematic review. Int J Drug Policy.

[57] Ahmed AO, Hunter KM, Mabe AP, Tucker SJ, Buckley PF (2015). The professional experiences of peer specialists in the Georgia Mental Health Consumer Network. Community Ment Health J.

[58] Mourra S, Sledge W, Sells D, Lawless M, Davidson L (2014). Pushing, patience, and persistence: peer providers' perspectives on supportive relationships. Am J Psychiatr Rehab.

[59] Jenkins GT, Shafer MS, Janich N (2020). Critical issues in leadership development for peer support specialists. Community Ment Health J.

[60] Ibrahim N, Thompson D, Nixdorf R, Kalha J, Mpango R, Moran G, Mueller-Stierlin A, Ryan G, Mahlke C, Shamba D, Puschner B (2020). A systematic review of influences on implementation of peer support work for adults with mental health problems. Soc Psychiatry Psychiatr Epidemiol.

[61] Haroun El Rasheed A, El Sheikh MM, El Missiry MA, Hatata HA, Ahmed N (2016). Addiction stigma among mental health professionals and medical students in Egypt. Addict Disord Treat.

[62] Walker G, Bryant W (2013). Peer support in adult mental health services: a metasynthesis of qualitative findings. Psychiatr Rehabil J.

[63] Watters JK, Biernacki P (1989). Targeted sampling: Options for the study of hidden populations. Soc Probl.

[64] Arpa S. Women who use drugs: Issues, needs, responses, challenges and implications for policy and practice. Background paper commissioned by the European Monitoring Centre for Drugs and Drug Adicction for Health and social responses to drug problems: A European guide. 2017. Retrieved from EuropeanResponsesGuide2017_BackgroundPaper-Women-who-use-drugs.pdf (europa.eu)

[65] Moran GS, Kalha J, Mueller-Stierlin AS, Kilian R, Krumm S, Slade M, Charles A, Mahlke C, Nixdorf R, Basangwa D, Nakku J (2020). Peer support for people with severe mental illness versus usual care in high-, middle-and low-income countries: study protocol for a pragmatic, multicentre, randomised controlled trial (UPSIDES-RCT). Trials.

[66] Puschner B, Repper J, Mahlke C, Nixdorf R, Basangwa D, Nakku J, Ryan G, Baillie D, Shamba D, Ramesh M, Moran G (2019). Using peer support in developing empowering mental health services (UPSIDES): background, rationale and methodology. Ann Glob Health..

